# A case report of syndrome of inappropriate antidiuretic hormone secretion with Castleman’s disease and lymphoma

**DOI:** 10.1186/1472-6823-13-19

**Published:** 2013-06-04

**Authors:** Chong-Gui Zhu, Qiu-Zi Zhang, Mei Zhu, Qiong-Li Zhai, Xiao-Yu Liang, Zong-Hong Shao, Emily C Ver Hoeve, Hui-Qi Qu

**Affiliations:** 1 Department of Endocrinology, Tianjin Medical University General Hospital, No 154 Anshan Road, Heping District, Tianjin, 300052, China; 2 Department of Pathology, Tianjin Medical University Cancer Hospital, Huan-Hu-Xi Road, Tiyuanbei, Hexin District, Tianjin, 300060, China; 3 Department of Surgery, Tianjin Medical University General Hospital, No 154 Anshan Road, Heping District, Tianjin, 300052, China; 4 Department of Hematology, Tianjin Medical University General Hospital, No 154 Anshan Road, Heping District, Tianjin, 300052, China; 5 Division of Epidemiology, Human Genetics and Environmental Sciences, University of Texas School of Public Health, 1200 Herman Pressler, Houston, Texas, 77030, USA

**Keywords:** Castleman’s disease, Hyponatremia, Lymphoma, Syndrome of inappropriate antidiuretic hormone secretion (SIADH)

## Abstract

**Background:**

Syndrome of inappropriate antidiuretic hormone secretion (SIADH) is a common cause of hyponatremia in hospitalized patients and is often described in patients with small-cell carcinoma of the lung. In this report, we described both Castleman’s disease and lymphoma coexisting in one patient with SIADH.

**Case presentation:**

A 70-year-old Chinese woman with a history of diabetes mellitus and insulin therapy had severe hyponatremia and gastrointestinal symptoms. Through a series of examinations, common causes such as pulmonary carcinoma were excluded. An abdominal mass was detected by computed tomography. Although the peripheral lymph node biopsy showed the pathological result as Castleman’s disease, the pathology of the abdominal lymph node revealed diffuse large B-cell lymphoma. After chemotherapy, the hyponatremia was treated during a period of follow-up.

**Conclusion:**

This patient presented with the rare clinical condition of inappropriate antidiuretic hormone secretion alongside Castleman’s disease and lymphoma. Asymptomatic hyponatremia may persist for some time suggesting that clinical physicians should pay attention to the mild cases of hyponatremia. We also hypothesized that Castleman’s disease is a condition of pre-lymphoma with both having the ability to cause SIADH. The possibility of lymphoma as well as Castleman’s disease triggering the development of SIADH should also be taken into consideration for conducting recurrent biopsies.

## Background

The syndrome of inappropriate antidiuretic hormone secretion (SIADH) is caused by the excessive release of antidiuretic hormone (ADH, also known as vasopressin) from the posterior pituitary gland [1,2] and can cause hyponatremia in hospitalized patients with an incidence as high as 30% [3]. SIADH is generally seen in patients diagnosed with small-cell lung cancer (SCLC) which was first described by Schwartz et al. in two patients with lung cancer [4].

SIADH is not difficult to diagnose in clinic. It can be secondary to a variety of disorders, such as medications, malignancies, surgery, and HIV infection or be idiopathic [1,2]. While SCLC is the primary malignancy causing SIADH, lymphoma can also induce this condition [5,6]. In this report, the diagnosis of Castleman’s disease was established before we suspected SIADH was caused by a malignancy. After extensive examinations, we made the final diagnosis of diffuse large B-cell lymphoma. Chemotherapy treatment was recommended for the patient and significantly ameliorated the SIADH symptoms.

## Case presentation

In October 2011, a 70-year-old Han Chinese woman with Type 2 diabetes (T2D) was admitted to Department of Endocrinology at the Tianjin Medical University General Hospital for uncontrolled hyperglycemia. The patient had a history of T2D for 21 years and received continuous long-term insulin treatment with a dosage of about fifty units per day. On the seventh day after admission, the patient developed symptoms of nausea and vomiting accompanied by left leg radiating pain without diarrhea and fever. She did not present with headache, dizziness, disturbance of consciousness, melana or hematemesis. The medications used in the hospital are listed in Table 1. The patient had no recent history of using diuretic agents. Serum sodium levels decreased to 112 mmol/l and chloride levels to 81 mmol/l with an effective osmolality at 267 mOsm/kg.H_2_O (normal range: 280–310 mOsm/kg.H_2_O). Urinary sodium increased to 85 mmol/l and chloride to 86 mmol/l with an osmolality of 257 mOsm/kg.H_2_O indicating hypotonicity during normal dietary salt intake. She was clinically normovolemic with no signs of fluid retention. Her hepatic and renal functions were normal with a serum creatinine of 68 (normal range: 44–115) umol/L. Fractional sodium excretion was calculated as 1.56%. Thyroid and adrenal function were measured with ACTH = 59.8 pg/mL(normal range: 0–46), cortisol = 25.6 ug/dL(normal range: 5–25), 24-hour cortisol in urine = 64.4 ug(normal range: 30–110), FT3 = 3.04 pmol/L(normal range: 3.5–6.5), FT4 = 19.76 pmol/L(normal range: 11.5–23.5), TSH = 3.657 uIU/mL(normal range: 0.3–5.0) and rT3 = 1.76 nmol/l (normal range: 0.43–1.15). The magnetic resonance imaging (MRI) of her pituitary gland showed herniation of the suprasellar cistern. Based on the MRI result, our first diagnosis was hypopituitarism, which was treated with intravenous hydrocortisone at 50 mg/day. Both the serum sodium and serum chloride increased from 112 to 116 mmol/l and from 81 to 84 mmol/l, respectively, on the following day.

**Table 1:** Medications used in the current hospitalization

**Medicine**	**Dose**	**Times**
Voglibose	0.3 mg	3
Glimepiride	4 mg	Once in the morning
Glimepiride	2 mg	Once in the evening
Insulin Aspart 30	28 IU	2
Irbesartan	150 mg	1

After further inquiry, the patient informed us of a past history of Mucosal-associated lymphoid tissue lymphoma (MALT) which had not received further treatment. We also discovered that she was last hospitalized on March 2011 for uncontrolled hyperglycemia without symptoms of nausea and vomiting. Nevertheless, hyponatremia had been noticed with serum sodium of 131 mmol/L and serum chloride of 95 mmol/l. Urinary investigation showed increased levels of sodium (90 mmol/l) and chloride (83 mmol/l) without corresponding osmolality data. Her renal function was normal with a serum creatinine of 63 umol/L. This information led us to believe that SIADH caused the patient’s hyponatremia. Three days after restricting fluid intake to 1000 ml/day, the serum sodium rose to 128 mmol/L from 116 mmol/L. Ten days later, the patient’s serum sodium levels increased to 142 mmol/l, and the symptoms of nausea and vomiting disappeared.

**Figure 1:**
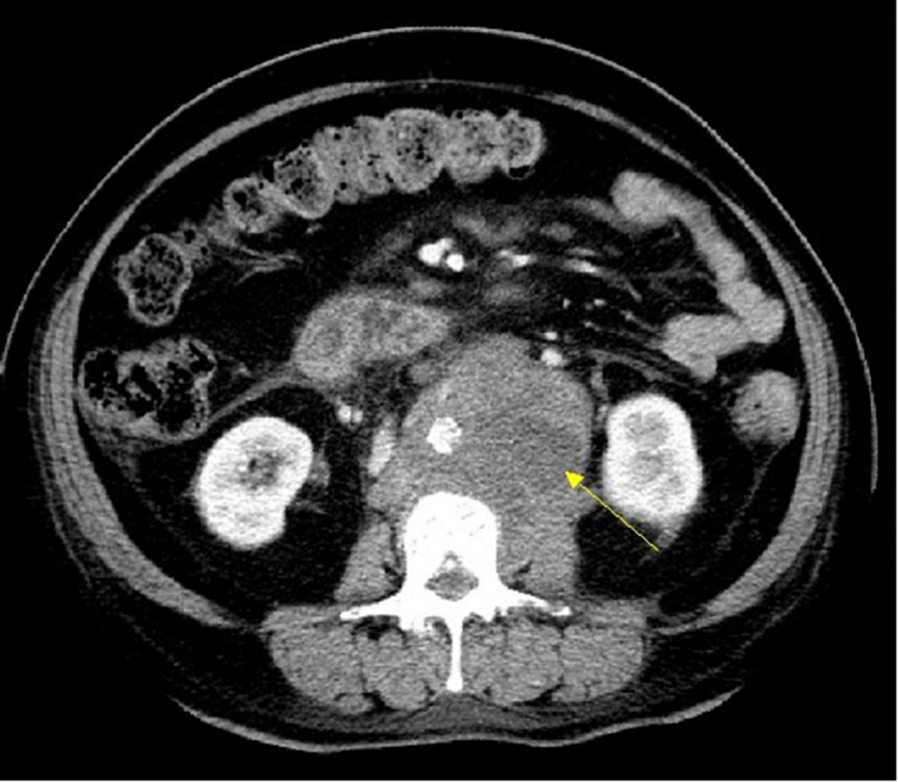
**CT scan of abdomen showing a mass.** Axial contrast-enhanced CT image shows a large lobulated irregular mass with inhomogenous enhancement that is encasing and anteriorly displacing the abdominal aorta.

**Figure 2:**
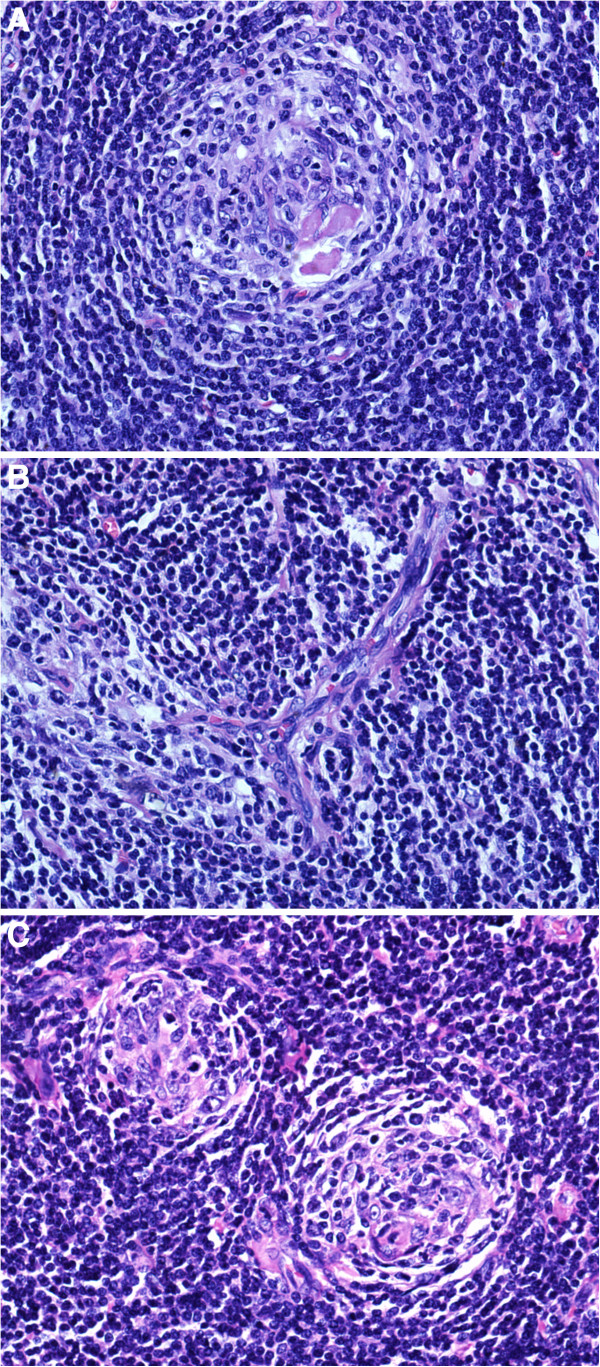
**The pathological exam suggests Castleman’s disease.** The gross examination of the inguenal lymph node biopsy specimen shows a fairly well-encapsulated, soft tan lesion with a largest dimension of 1.2 cm. Microscopy reveals areas of hyaline-vascular Castleman’s disease-like pattern. The majority of the lymphoid follicles had atrophic or regressive germinal centers, and some of them are penetrated by capillaries. There was concentric layering of lymphocytes in an onion-skin appearance and one or more penetrating blood vessels. The interfollicular stroma was also prominent with numerous hyperplastic vessels of the post-capillary venule type and plump endothelial lining. (**A**) concentric layering of lymphocytes in an onion-skin appearance; (**B**) a “lollipop” appearance; (**C**) two atrophic germinal centers within a single mantle zone.

Since the patient had a past history of MALT, we performed a gastric endoscopy, which indicated the absence of lymphoma. Additionally, the serology result was negative for purified protein derivative (PPD), and the serum and urine protein electrophoresis were also negative for monoclonal gammopathy. Additional laboratory findings were listed in Table 2. A negative chest computed tomography (CT) scan excluded the possibility of SCLC as the cause of SIADH in this patient. However, we noticed that the patient had obvious pain in the left leg, abdominal distension, and skin itching on a clinical exam and further discovered that her abdomen circumference had increased significantly in the last six months. An abdominal CT scan showed that a mass measuring approximately 49 mm*70 mm*90 mm was located on front lumbar vertebra 1–4 and the surrounding abdominal aorta. A subsequent abdominal enhancement CT revealed that the mass might be lymphoma (Figure 1). As the pathological result was critical for her diagnosis, we conducted a left inguinal lymph node puncture showing no abnormal lymphocytes. Consequently, a whole left inguinal (2 × 1 × 0.5 cm) lymph node was resected. The pathological morphology revealed that the lymph node structure was replaced with substantial amounts of lymphoid tissue and fibrosis. Thus, the diagnosis of Castleman’s disease was established according to the pathological exam (Figure 2A, B, C).

**Table 2:** Laboratory findings in the current hospitalization

**Exam**	**Value**	**Reference range**	**Unit**
HbA1c	10.50%	4-6%	
FBG	12.5	3.9-6.1	mmol/L
24-h proteinuria	124.2	0-150	mg
Blood urea nitrogen	4	1.7-8.3	mmo/L
Serum uric acid	144	140-414	μmol/L
Serum creatinine	68	44-115	μmol/L
Ccr	86.8	80-120	ml/min
ACTH	59.8	0-46	pg/mL
Cortisol	25.6	5-25	μg/dL
24-hour cortisol in urine	64.4	30-110	ug
FT3	3.04	3.5-6.5	pmol/L
FT4	19.76	11.5-23.5	pmol/L
TSH	3.657	0.3-5.0	μIU/mL
rT3	1.76	0.43-1.15	nmol/l
ESR	21	0-20	mm/h
Lactic acid dehydrogenase	186	94-250	U/L
ß2-microglobulin	2.04	0.8-2.0	mg/L
Antinuclear antibody	Negative		
Immunoglobulin G	811	751-1560	mg/dl
Immunoglobulin A	64.8	82-453	mg/dl
Immunoglobulin M	48.1	46-304	mg/dl
C3	88.9	79-152	mg/dl
C4	17.10.	16-38	mg/dl
C-reactive protein	1.07	<0.80	mg/dl
Circulating immunologic complex	4.2	<13	U/ml
Immunoglobulin E	<5.00	<165	IU/ml
Alpha Fetoprotein	4.42	0-20	ng/ml
Ferritin	272.87	4.6-204	ng/ml
Carcinoembryonic antigen	1.42	0-5	ng/ml
Carbohydrate antigen19-9	<0.60	0-37	U/ml
Carbohydrate antigen 242	0.05	0-20	U/ml
Carbohydrate antigen 153	16.3	0-30	U/ml
HIV	Negative		

**Figure 3:**
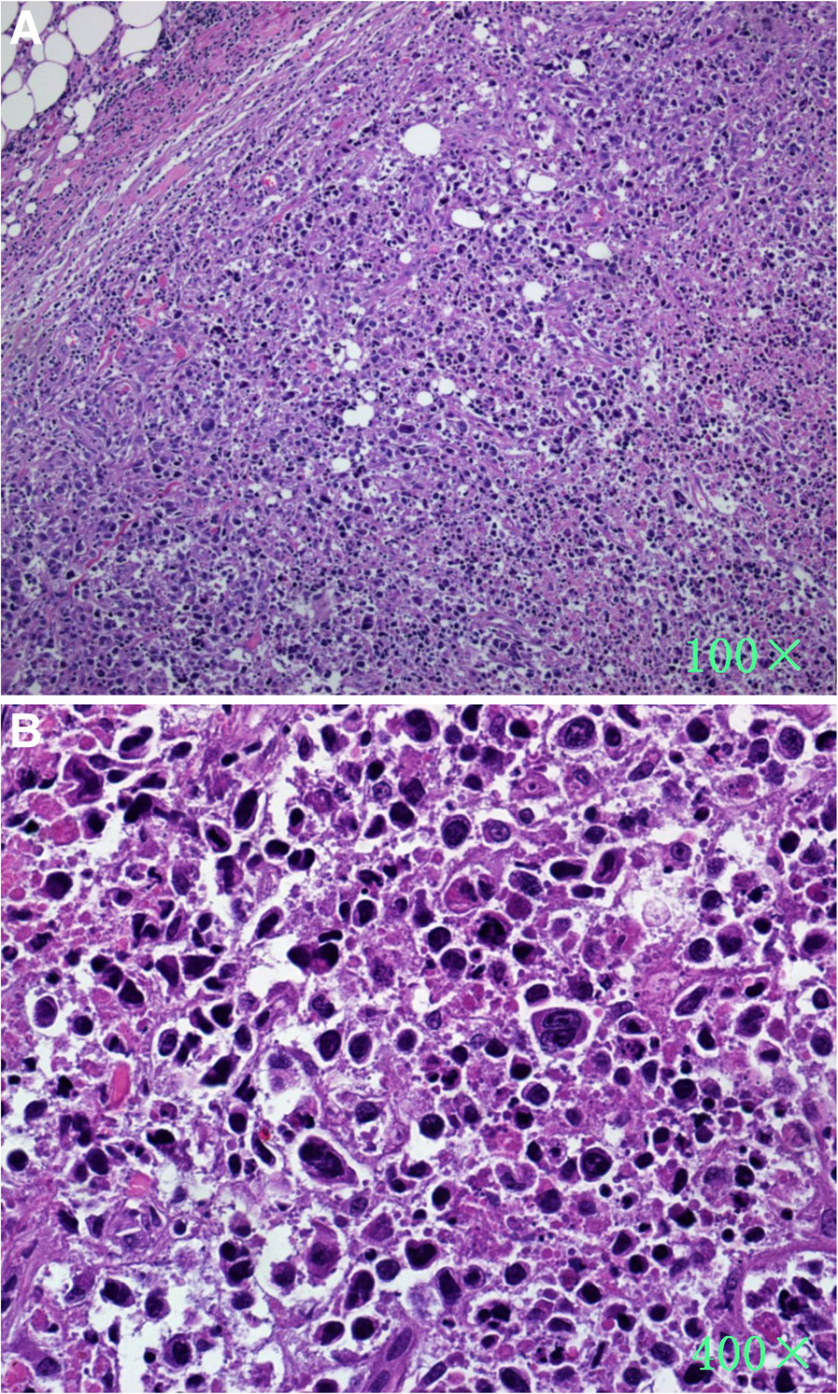
**An abdominal lymph node biopsy suggests the diagnosis of diffuse large-B cell lymphoma, anaplastic subtype.** (**A**) At low magnification, the normal nodal architecture was totally effaced and the node was diffusely infiltrated by a population of large atypical lymphocytes with a highly characteristic morphology. Extensive infiltration of the perinodal adipose tissue was observed as well. (Haematoxylin and eosin). (**B**) High magnification show that nuclei were round to oval with fine chromatin; Extensive areas of coagulative necrosis and karyorrhectic nuclei were present. Reed-Sternberg–like forms and giant cells are also prominent and conspicuous nucleoli are seen in these cells. Eosinophilic region was seen near the nucleus, probably representing a prominent Golgi apparatus.

Although hyponatremia was corrected, her hemoglobulin levels decreased from 109 g/L to 96 g/L. With her consent, we conducted a biopsy of the enlarged abdominal lymph node by using a celioscope. The pathological exam disclosed diffuse large B-cell lymphoma with an anaplastic subtype (Figure 3A, B).

After the diagnosis of lymphoma was established, the patient was transferred to the Department of Hematology for further treatment. For the etiology treatment of B-cell lymphoma, the chemotherapy of cyclophosphamide, hydroxydaunorubicin, oncovin and prednisone (CHOP) with rituximab (a monoclonal antibody against the protein CD20) was administered. The patient’s serum sodium level stabilized without fluid restriction.

## Discussion

In this case, the hyponatremia had existed for a long time and failed to gain proper attention until serious clinical manifestations developed. Her past history of MALT as well as the success of fluid restriction and failure of hydrocortisone infusion suggested the possibility of hyponatremia caused by SIADH. One limitation of our investigation was the failure to measure urine uric acid and urea levels. Fractional uric acid excretion (FE-UA) has been demonstrated as a useful criterion in the diagnosis of SIADH particularly when attempting to differentiate between SIADH and hypovolaemic hyponatremia [7].

The main therapeutic issue in SIADH is fluid excess, and hyponatremia is dilutional in essence. Fluid restriction is the primary treatment option for SIADH. The vasopressin V2 receptor antagonist is effective for assisting the SIADH treatment [8]. Urea administration could be used in patients with chronic SIADH but also in acute SIADH like in critically ill patients [9].

Once a diagnosis of SIADH is established, determining its cause is most important. Castleman’s disease is characterized by non-cancerous growths that may develop in the lymph node tissue throughout the body [10]. Lesions often occur in the chest, abdomen, or neck, where the abnormal enlargement of lymph nodes could usually be found. Although SIADH is reported to be associated with Castleman’s disease [11], the possibility of general lymphoma in this case could not be excluded. Several major points supported this conclusion including: (1) past history of MALT; (2) occurrence of itching (characteristic symptom of lymphoma); (3) CT scan findings suggested lymphoma; and (4) the manifestation of progressive anemia. Moreover, the patient had a long history of T2D treated with insulin. Recent studies suggest that insulin treatment may be associated with an higher risk of the developing certain types cancers including lymphoma [12].

Although Castleman’s disease is inherently a nonneoplastic process, an association with concurrent or subsequent lymphoma has been well described [13]. The hyaline-vascular type of Castleman’s disease which contains numerous lymphoid follicles should be differentiated from Hodgkin lymphoma and low-grade B-cell lymphomas [14]. Several B-cell lymphomas with prominent atrophic germinal centers and hyaline vascular penetration including follicular lymphoma, mantle cell lymphoma, and nodal marginal zone lymphoma tend to be misdiagnosed as Castleman’s disease due to the similar clinicopathologic features [15]. Furthermore, multicentric Castleman’s disease could show similar FDG PET/CT appearance mimicking lymphoma, which reveals that Castleman’s disease and lymphoma exhibit similar functions [16]. After consulting the literaure and examining the presentation of SIADH caused by both diseases, we hypothesized that Castleman’s disease can develop into lymphoma, and a variety of pathological lymphomas may result in the development of SIADH [6,17]. One possible mechanism is the effect of hypercytokinemia. Some cytokines, such as interleukin (IL)-2, IL-6, IL-1β, and tumor necrosis factor (TNF)-α, have been reported to stimulate parvicellular and magnocellular neurons to secrete more ADH thus causing SIADH [18,19].

In our case, three separate biopsies showed differing results suggesting that this procedure is so imperative for certain conditions that several biopsies may be needed for a final diagnosis.

## Conclusion

This report describes a rare case, in which an older woman had both Castleman’s disease and lymphoma coexisting with SIADH. We wish to highlight the following key points: First, lymphoma as well as Castleman’s disease, though uncommon, is an important cause of SIADH, and mild hyponatemia should get proper attention by clinical endocrinologists. Second, repeated lymph node biopsy may be crucial for the etiological diagnosis of SIADH.

## Consent

Written informed consent was obtained from the patient for publication of this case report and the accompanying images. A copy of the written consent is available upon request for review by the Journal Editor.

## Abbreviations

SIADH: Syndrome of inappropriate antidiuretic hormone secretion; ADH: Antidiuretic hormone; ACTH: Adreno-corticotrophic hormone; IL: Interleukin.

## Competing interests

The authors declare that they have no competing interests.

## Authors’ contributions

MZ and CGZ led the conception and design, acquisition of data, review of literature, and drafted the manuscript. ZHS reviewed the manuscript. HQQ contributed the concept of research paper and critically reviewed the manuscript. QLZ expatiated the pathology and provided the pictures. All authors read and approved the manuscript.

## Authors’ information

MZ is the director of the Department of Endocrinology, Tianjin Medical University General Hospital. CGZ is the resident of Department of Endocrinology. HQQ is an Assistant Professor, Division of Epidemiology, Human Genetics and Environmental Sciences at School of Public Health, University of Texas Health Science Center at Houston.

## Pre-publication history

The pre-publication history for this paper can be accessed here:

http://www.biomedcentral.com/1472-6823/13/19/prepub
